# Visual-Constructional Ability in Individuals with Severe Obesity: Rey Complex Figure Test Accuracy and the Q-Score

**DOI:** 10.3389/fpsyg.2017.01629

**Published:** 2017-09-20

**Authors:** Hanna L. Sargénius, Frederick W. Bylsma, Stian Lydersen, Knut Hestad

**Affiliations:** ^1^Department of Psychology, Faculty of Social Sciences and Technology Management, Norwegian University of Science and Technology Trondheim, Norway; ^2^Centre for Old Age Psychiatric Research, Innlandet Hospital Trust Ottestad, Norway; ^3^Neuropsychological Services PC Chicago, IL, United States; ^4^Regional Centre for Child and Youth Mental Health and Child Welfare, Norwegian University of Science and Technology Trondheim, Norway; ^5^Department of Research, Innlandet Hospital Trust Brumunddal, Norway; ^6^Department of Public Health, Hedmark University of Applied Sciences Elverum, Norway

**Keywords:** obesity, visual-constructional ability, organizational strategy, Q-score

## Abstract

The aims of this study were to investigate visual-construction and organizational strategy among individuals with severe obesity, as measured by the Rey Complex Figure Test (RCFT), and to examine the validity of the Q-score as a measure for the quality of performance on the RCFT. Ninety-six non-demented morbidly obese (MO) patients and 100 healthy controls (HC) completed the RCFT. Their performance was calculated by applying the standard scoring criteria. The quality of the copying process was evaluated per the directions of the Q-score scoring system. Results revealed that the MO did not perform significantly lower than the HC on Copy accuracy (mean difference −0.302, CI −1.374 to 0.769, *p* = 0.579). In contrast, the groups did statistically differ from each other, with MO performing poorer than the HC on the Q-score (mean −1.784, CI −3.237 to −0.331, *p* = 0.016) and the Unit points (mean −1.409, CI −2.291 to −0.528, *p* = 0.002), but not on the Order points score (mean −0.351, CI −0.994 to 0.293, *p* = 0.284). Differences on the Unit score and the Q-score were slightly reduced when adjusting for gender, age, and education. This study presents evidence supporting the presence of inefficiency in visuospatial constructional ability among MO patients. We believe we have found an indication that the Q-score captures a wider range of cognitive processes that are not described by traditional scoring methods. Rather than considering accuracy and placement of the different elements only, the Q-score focuses more on *how* the subject has approached the task.

## Introduction

**Figure 1:**
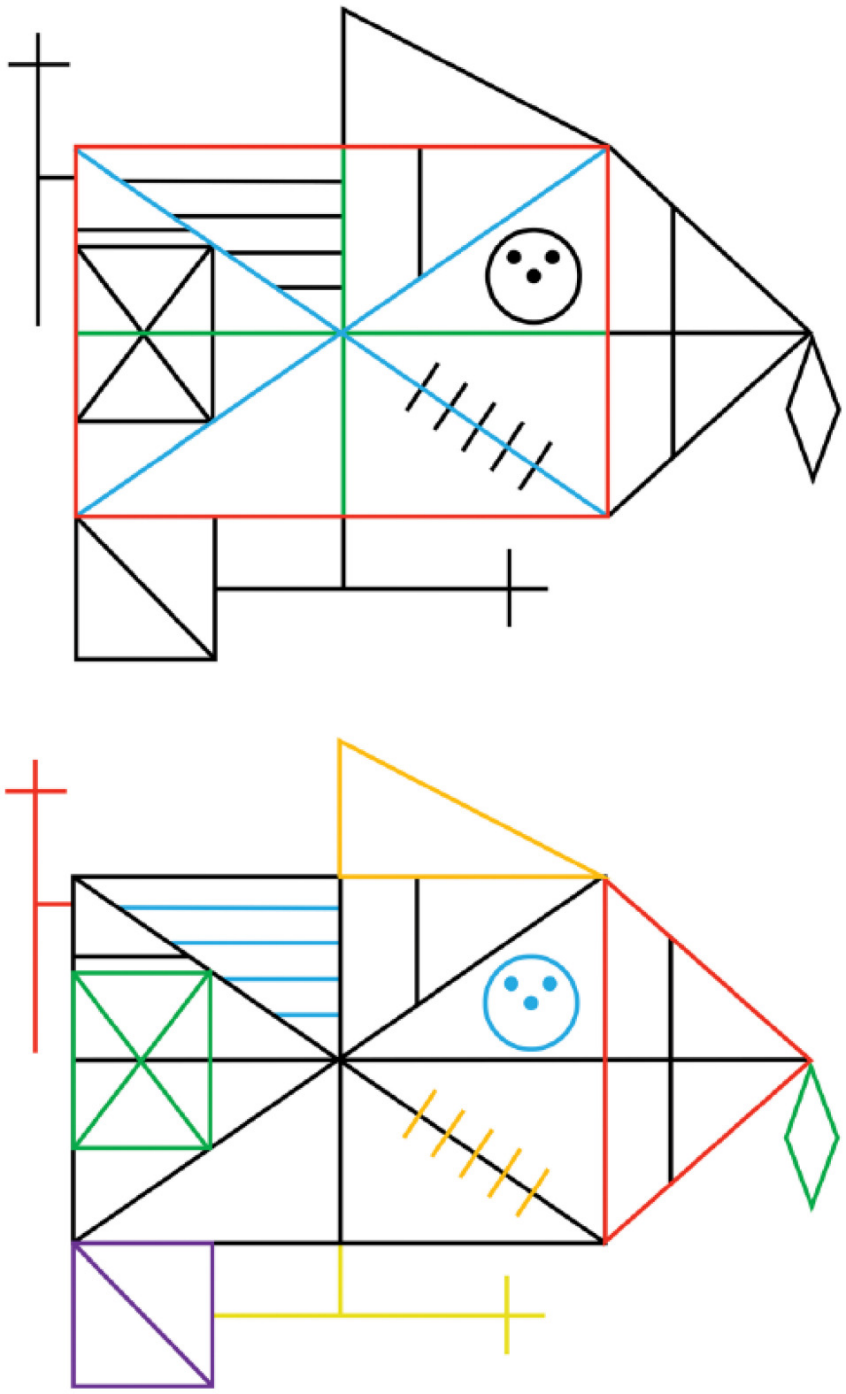
Illustrations from Weider et al. (2016) demonstrating the scoring of the Q-score, based on the original RCFT. **(Top)** Of the 3 units considered most important within the Q-Rey scoring system, each one is awarded both unit and order points. **(Bottom)** the 10 remaining units within the Q-Rey scoring system, each only awarded by unit points.

The Rey-Osterrieth Complex Figure Test (RCFT) has become a widely used test when assessing visuospatial constructional abilities for clinicians and researchers alike. The test, which was developed by Rey (1941) and standardized by Osterrieth (1944)—with the original manuscript later translated into English by Corwin and Bylsma (1993)—provides valuable information on such cognitive processes as perceptual organization, visuospatial constructional ability, and visual memory (Strauss et al., 2006; Lezak et al., 2012). The RCFT employs a complex geometrical figure as the stimulus; it comprises a large rectangle with horizontal and vertical bisectors, two diagonals, and additional geometric details (Figure 1). The test proceeds with the subject being asked to draw a copy of the presented figure as accurately as possible. This task is followed by a distraction and a request to redraw the same figure from memory. The distraction and recall sequence is repeated twice, with designated delays in between, depending on the administration chosen.

One of the fundamental aspects of accurately completing the RCFT is organizing the stimulus figure into meaningful perceptual units while copying it (Deckersbach et al., 2000). In addition, having a coherent drawing style with a balance between efficiency and attention to detail (Rose et al., 2014) enhances the subsequent free recall of the figure from memory (Savage et al., 1999, 2000). The ideal approach when starting the figure copy is to use the global, external features and to draw each one continuously (Bylsma, unpublished manuscript). The opposite would be to rely on the local, internal details in the construction process. That approach is thought to be more fragmented and inefficient, thereby leading to poor visual memory for the figure (Rose et al., 2014). From a clinical standpoint, a fragmented organizational approach is often interpreted as an indication of cognitive impairment (Lezak et al., 2012).

There is increased interest in the diverse effects that obesity seems to have on cognition. Several epidemiological reports have demonstrated that especially midlife obesity contributes to the development of Alzheimer's disease (AD) (Gustafson et al., 2004a,b, 2012; Kivipelto et al., 2005; Tolppanen et al., 2014). The link between obesity and AD is suggested to be mediated by obesity-related comorbidities like diabetes and hypertension. Hypertension, for instance, may increase the risk by that it induces small-vessel disease and white-matter lesions (Skoog et al., 1996). Recent studies have found evidence for the obesity-dementia association to potentially be independent, however (Buchman et al., 2005; Whitmer et al., 2005, 2007, 2008; Profenno et al., 2010). To complicate things even more, underweight in midlife or a decrease in BMI from midlife into late-life, appear to function as risk factors for late-life AD as well (Tolppanen et al., 2014; Qizilbash et al., 2015). In cases where AD has been diagnosed, higher levels of body mass seem protective by slowing down cognitive decline, if being close to the onset of dementia (Luchsinger et al., 2007; Gustafson et al., 2009). It may look as if these are discrepant findings, however, this only illustrates that the link between body mass and cognitive decline is not straightforward, but rather that a U-shaped relationship may exist between the two (Gustafson et al., 2003; Stewart et al., 2005; Anstey et al., 2011). Traditional measures for adiposity, such as BMI with its categorical cut-offs, is somewhat arbitrary as an individual's body composition changes with increasing age (Luchsinger and Mayeux, 2007). Subsequently, instead of considering level of BMI at a single time point, the lifespan trajectories of adiposity itself is of greater importance (Gustafson and Luchsinger, 2013). Additionally, many who develop dementia have combinations of Vascular dementia and AD, which also must be taken into consideration. In a study of primary prevention of AD, Norton et al. (2014) have pointed at 7 potential risk factors for this disease to target; diabetes, midlife hypertension, midlife obesity, physical inactivity, depression, smoking, and low educational attainment. Whether the various components operate independently of each other or in combination, they all appears to be risk factors for impairments of the brain and dementia.

In neurodegenerative diseases like AD, visuospatial dysfunction is one of the symptoms that can occur (Possin, 2010). Various degenerative neural diseases cause confined atrophy to neural networks, however, characteristic of the disease in question. Consequently, the type of disease affects visuospatial processing in a dissimilar and particular manner (Possin, 2010). Considering the suggested link between neuro-degeneration to the brain and obesity, visuospatial ability could potentially be affected in a way that is distinctive of obesity. Furthermore, if visuospatial constructional inefficiency is common to obese individuals, this could be a prodigious primer to poor visual (non-verbal) memory, a domain that appears to be affected in this population (Boeka and Lokken, 2008; Cheke et al., 2016; Sargénius et al., 2017). Few studies have investigated visual-constructional ability specifically among obese individuals. Boeka and Lokken (2008) and Lokken et al. (2010) have found a relationship between obesity and poorer performance on the RCFT, independent of comorbidities such as hypertension, diabetes, and obstructive sleep apnea (Boeka and Lokken, 2008). In contrast, Fergenbaum et al. (2009) was not able to find any effect for obesity on performance on the Clock Drawing Test in their study. In another study, in which results from the RCFT and The Group Embedded Figure Task (GEFT) were combined, Roberts et al. (2007) discovered medium-to-large effect sizes for both tests, although in opposite directions. A limitation to this study is the small and unequal sample sizes between the normal-weight and overweight groups. Nonetheless, the results are intriguing.

Considering the small number of studies that has been conducted, and the inconsistency in which comorbidities that is controlled for, it is not possible to draw any conclusions yet whether there exist an independent association between impaired visuospatial constructional ability and obesity (Prickett et al., 2015). In contrast, an extensive body of research seems to confirm the presence of a constructional ability impairment and reduced visual memory among individuals with Anorexia nervosa (AN) (Lopez et al., 2008a,b; Lang et al., 2014). This has to some degree been confirmed among subjects suffering from Bulimia nervosa (BN) as well (Lopez et al., 2008c; Darcy et al., 2015) although findings have been more varied than for those with an AN diagnosis.

In a previous study in which we applied the RCFT, we found no evidence for worse performance on the copy portion of the RCFT among the severely obese patients that were studied (Sargénius et al., 2017). We did find, however, this group to perform significantly worse on the cognitive domain of visual memory. Therefore, we proposed that the organizational strategy demonstrated by obese patients when completing a complex figure task, and the nature of the errors they make when completing this task, could be characteristic of the neuropsychological pathology observed in obese patients. Important information could be lost when only traditional RCFT scoring criteria are used, unaccompanied by other measures. Lang et al. (2014) and Lopez et al. (2008a) have advanced a similar argument regarding patients suffering from restrictive disorders. The RCFT with its scoring criteria is renown and widely-used in the neuropsychological milieu, but obesity in clinical terms is a relatively new condition, and we do not know if we have the right tools and methodologies at hand at this moment. The qualitative approach to the task made by anorexic and bulimic patients seems to follow a continuum, with the anorexic patients more cognitively impaired than the bulimic patients (Weider et al., 2016). Both patient groups do perform worse than normal-weight individuals, however. This finding could suggest that extreme weight conditions—with restrictive eating disorders and morbid obesity at opposite ends of an eating disorder spectrum—have similar executive dysfunction profiles (Fagundo et al., 2012; Weider et al., 2016). A valuable approach to understanding the effects of obesity possibly will be provided if MO patients and anorexic/bulimic patients were placed on the same continuum regarding the cognitive abilities essential for RCFT performance.

Different systems assessing for qualitative aspects of visuospatial task performance, each varying as a function of the qualitative features evaluated, have frequently been applied within the eating disorder literature. The qualitative scoring method proposed by Booth (2006), later investigated more closely by Lopez et al. (2008a) and Rose et al. (2014), has been the prevailing system in this research area despite this system's short history. It provides an index of central coherence (the tendency to concentrate on details rather than on global or gestalt features), composed of an Order of construction Index and a Style Index. This scoring system, however, has by some been criticized because of its complexity, and as such, lessening its clinical value (Weider et al., 2016). The critics have argued that a more convenient measure is needed. Weider et al. argues that: “*although the order in which the different units of the figure are completed is important to evaluate as part of the cognitive style related to weak central coherence*, [this system] *is too heavily based on this dimension at the expense of scoring the identification of the gestalts drawn as an unbroken unit*.” The Q-score system (Bylsma et al., 1995; Bylsma, unpublished manuscript) has been proposed to be a good alternative to the Central Coherence Index. Considering that the introduction of the Q-score dates back to 1995, in the eating disorder literature this system is the “new kid in the block.” A key strength to the Q-score system is that it awards points for identifying the basic components or gestalts of the figure, and it is easy for the clinician to use, yet it provides a wide score range that can be benefit from when evaluating the patient's abilities. A weakness to many qualitative scoring systems is that they offer a range of scores that is too narrow, which in turn produces heavily skewed distributions when they are applied in healthy populations (Wilson and Batchelor, 2015). Scores derived from the Q-score, however, is found to be approximately normally distributed when applied in healthy samples, and reveal moderate variability between subjects (Troyer and Wishart, 1997; Wilson and Batchelor, 2015). In clinical use, it has already demonstrated a robust effect in anorexic and bulimic patients, even after statistical adjustments, especially relating to nadir body mass index (Weider et al., 2016). This finding suggests that the Q-score may be a good measure for capturing the presence of a stable cognitive difficulty among eating disorder patients. If the Q-score can capture the quality of an eating-disordered individual's approach to the RCFT copy, independent of whether the patient is at the lower (anorexic) or upper (obese) end of the body mass spectrum, then this system could have clinical utility in the assessment of eating disordered patients.

As far as we know, only one study (Roberts et al., 2007) has applied a somewhat qualitative approach when analyzing RCFT performance among obese individuals. Their interpretation of performance quality among the overweight subjects was based mainly upon the combined performance scores derived from the RCFT and The Group Embedded Figure Task (GEFT: Witkin et al., 1971), however—not merely the RCFT alone—and with no standardized qualitative scoring system in place. Furthermore, no researchers have applied the Q-score in the context of obesity. The objectives of this study are (1) to investigate the utility of the Q-score in a sample of non-demented MO patients and (2) to compare this qualitative scoring system to the traditional scoring guidelines for determining RCFT performance accuracy and efficiency.

## Materials and methods

### Participants

Ninety-six non-demented MO patients (74 females) who were referred to weight-loss treatment at Innlandet Hospital Trust completed the RCFT as part of a larger battery of neuropsychological tests (see Table 1). The test battery is described in more detail elsewhere (Sargénius et al., 2017). The inclusion criteria were that the patient had to be eligible and accepted for weight-loss treatment according to health care guidelines—being between 18 and 60 years of age, and having ≥ 30 BMI points. Participants with a past or current history of (a) neurological disorder or injury (e.g., dementia, stroke, or seizures), b) moderate or severe head injury (defined as >10-min loss of consciousness), (c) severe psychiatric illness (e.g., schizophrenia and bipolar disorder), (d) alcohol or drug abuse, e) learning disorder, or f) developmental disabilities were not enrolled into this study.

**Table 1:** Neuropsychological test battery.

**Cognitive domain**	**Test variables**
General functioning	Wechsler Adult Intelligence Scale III (WAIS-III)
	(Picture completion, Vocabulary, Digit symbol, Similarities, Block design, Matrix reasoning, Information)
Verbal learning and memory	California Verbal Learning Test II (CVLT-II)
	Wechsler Memory Scale Revised (WMS-R) (logical memory I and II)
Visual learning and memory	WMS-R (visual memory I and II)
	RCFT (immediate recall trial, delayed recall trial)
Speed of information processing	Trail Making Test (TMT) A
	WAIS-III (digit symbol)
	Color Word Interference Test (CWIT)
	(color naming, word reading)
Visuospatial ability	WAIS-III (block design, matrix reasoning)
	RCFT (copy)
Working memory	Paced Auditory Serial Addition Test (PASAT)
	(3 s, 2 s)
	WMS-R (spatial span forward and backwards)
Executive function	Category Test
	Wisconsin Card Sorting Test (WCST)
	D-KEFS Tower Test
	TMT B
	CWIT (inhibition, inhibition/switching)
Verbal fluency	D-KEFS Verbal Fluency Test
Attention and vigilance	Conners' Continuous Performance Test II (CPT-II)
Motor function	Grooved Pegboard Test
	Dynamometer (grip strength)

*Test battery used in the neuropsychological assessment in Sargénius et al. (2017)*.

All patients were screened for depressive symptoms with the Beck Depression Inventory II (BDI-II). Depression was treated as a dichotomous variable in the analyses, with patients divided into those with no or mild depression (BDI-II < 11) vs. those with mildly depressive to severely depressive symptoms (BDI-II > 11). To secure the measurements to become as sensitive as possible to associations that are already weak when dealing with small sample sizes, a cut-off on mild depressive symptoms was chosen instead of a cut-off at borderline clinically depressed (BDI-II > 17). See Table 2 for descriptive statistics.

**Table 2:** Descriptive statistics for the MO and HC-group.

	**MO (*n* = 96)**			**HC (*n* = 100)**		
	**Mean (SD)**	**Min**	**Max**	**Mean (SD)**	**Min**	**Max**
Sex (male/female)	22/74			44/66		
Age	43.55 (9.25)	18	60	41.51 (11.37)	20	60
Education	13.13 (2.63)	8	22	13.91 (2.99)	7	20
BMI	42.61 (5.52)	31.8	59.7			
WC	127.11 (13.78)	98	164			
BDI-II	10.0 (9.22)	0	38			
SBP	139 (15.58)					
DBP	85.9 (8.46)					

*BMI, Body Mass Index; WC, waist circumference; BDI-II, depression; SBP, systolic blood pressure; DBP, diastolic blood pressure; available for MO only*.

The healthy controls (HC) were a sample of 100 subjects (66 females) from Baltimore, Chicago, and Toronto areas. HCs were drawn from a pool of 417 subjects, selected to be within the same age range as the patients (18–60 years), and with minimum 7 years of education. No information on weight and height was available for HCs, who were all participants in the control groups of other research studies (Baltimore Longitudinal Study of Aging; ABC Study), and considered healthy, normal-functioning adults. They had no evidence of cognitive or mental status decline, based on their scores on the Mini Mental State Examination [MMSE: Folstein et al., 1975; mean (SD) 28.79 (1.19)] scores.

### Material

The RCFT was administered in the standard manner (Meyers and Meyers, 1995). Performance accuracy was calculated by applying the standard scoring criteria, in which the geometrical figure is divided into 18 units and scored on a 2-point scale for both accuracy and placement (Meyers and Meyers, 1995).

The quality of the copying process was evaluated per directions of the Q-score scoring system (Bylsma, unpublished manuscript). The Q-score is based on the assumption that in order to copy the figure most efficiently, the subject should draw the basic structural components first, and then add the details in the most efficient manner. The term “efficiently” refers to completing the figure using the least number of lines possible (a minimum of 42 lines, whether straight, a circle or a dot), and in appropriate sequence. The stimulus figure comprises 13 discrete units, some structural and others placed on the primary structural elements. Drawing the main structural units initially, using sequential lines (each unit comprises two or more lines) is considered most important, and doing so achieves points for accuracy and for order of drawing (see Table 3 for unit definition and scoring criteria). To achieve order points, however, the base rectangle must be completed first, followed by the bisectors and diagonals; which of these latter units is drawn second and which is drawn third is irrelevant (See Figure 1). Thereafter, accuracy points are awarded only for details drawn as units (consecutive lines). Any mark the subject makes in their effort to complete any aspect of the figure is considered an additional line, and therefore are counted and numbered as a line used to complete the figure. This includes any modifications of existing previously drawn lines, overdrawing a line, erasing a line, close a gap between two lines etc. (Bylsma, unpublished manuscript).

**Table 3:** Unit scores and Order scores for elements of RCFT within the Q-score scoring system.

**Unit**	**Unit points**	**Order points**	**Scoring criteria**
Base rectangle	3	3	Must be completed in four consecutively numbered lines. If completed as lines 1–4, score 3 points for Order. No other combination of lines is awarded Order points for this Unit.
Diagonals	2	2	Top left to bottom right of the Base Rectangle and Top right to bottom left of the Base Rectangle completed as 2 consecutively numbered lines. If completed as lines 5 and 6, 6 and 7, or 7 and 8, score 2 points for Order.
Bisectors	2	2	Vertical midline (top to bottom of Base Rectangle) and horizontal midline (left to right side of Base Rectangle) completed as 2 consecutively numbered lines. If completed as lines 5 and 6, 6 and 7, or 7 and 8, score 2 points for Order. ^*^
Box with diagonals	1		Completed as no more than 5 consecutively numbered lines. ^**^
Left cross	1		Completed as 3 consecutively numbered lines
4 horizontal lines	1		Completed as 4 consecutively numbered lines (all 4 must be present) ^*^
Square on bottom	1		Completed as no more than 4 consecutive lines. ^**^
Triangle at right	1		Completed as 2 consecutively numbered lines. ^**^
Top triangle	1		Completed as 2 consecutively numbered lines. ^**^
Circle with three dots	1		Circle and 3 dots completed sequentially. ^*^
5 crosshatch lines	1		Completed as 5 consecutively numbered lines (all 5 must be there). ^*^
Diamond	1		Completed as 4 consecutively numbered lines
Bottom cross	1		Completed as 3 consecutively numbered lines. ^**^

*The three units considered most important within the Q-Rey scoring system (the base rectangle, the diagonals, and the bisectors) are the only units that are awarded points based on order of completion in addition to ordinary unit points. The 10 remaining units are awarded unit scores only*.

** *Exception is given from the scoring criteria under certain circumstances. Please confer the test manual for information on which exceptions that apply*.

* *Important note to the administrator concerning the scoring for this Unit. Please confer the test manual*.

The Q-score is calculated for the copy trial only, which comprises unit and order points. Maximum score in Order is 7 points, and on the Unit the maximum score is 17 points. Higher scores indicate a more efficient planning and strategic approach for making the copy. To illustrate, as mentioned previously, the base rectangle must be completed first, followed by the bisectors and diagonals. According to Bylsma (unpublished manuscript), these three units should ideally be completed in 8 consecutively numbered lines. Therefore, to be awarded maximum score in Order points, all the elements must be completed using lines numbered less than 9. The rest of the stimulus figure are just assigned Unit points, not Order points, but only if they are completed within the respective number of lines that has been assigned to that specific element (see Table 3). To ensure that the Q-score was calculated correctly for the MO patients, participants were videotaped while completing the RCFT, and the videos were transcribed such that the sequence of the subject's copy production was generated with all lines sequentially numbered in the order completed by the subject.

Compared to other qualitative scoring systems that often have the tendency to reveal heavily skewed distribution scores, the Q-score distribution is approximately normal when applied in healthy populations (Troyer and Wishart, 1997; Wilson and Batchelor, 2015).

### Ethics

All MO patients were seeking weight-loss treatment voluntarily, and treatment was offered based upon the referral by the patient's primary care physician, in combination with a professional evaluation of medical records at the hospital. The patients were informed that their participation and performance in this research would have no implications for their treatment. Written informed consent from all participants was obtained. Ethical approval was received in July 2012 from the Regional Committee of Medical and Health Research Ethics (REC), Norway: Reference 2012/966.

### Statistical analysis

The sociodemographic variables were compared using Pearson's chi-squared test for gender, and Student's *t*-test for age and education. Pearson's correlation was used to examine the associations among sociodemographic, clinical factors, and RCFT performance. Linear regression analyses, with the summary score as the dependent variable and group as a covariate, were used to analyse differences between the groups. This analysis was performed unadjusted and adjusted for gender, age, and education. The Q-score for the copy trial was then converted into age-appropriate T-scores, by using the HCs as reference. The T-scores were calculated by applying a “sliding groups” technique, whereby some of the same HCs are used in 2 age groups to obtain average performance scores across sequential 5-year age spans. First, Mean and SD values for different average age groups were generated at 5-year intervals, with the Mean and SD computed over 10-year age spans (e.g., for 30-year-olds, the mean and SD are computed based on patients between age 25 and 35; for 35-year-olds, Mean and SD are computed based on HCs between age 30 and 40, etc.). The appropriate age group Mean and SD was used for each patient to compute the patient's T-score. Normality of all relevant cognitive variables residuals was confirmed by visual inspection of Q-Q plots. Two-tailed *p*-values of < 0.05 were used to indicate statistical significance. Ninety-five per cent confidence intervals (CI) are reported where relevant. SPSS 24.0 (IBM Corporation, Armonk, NY, USA) was used in all statistical analysis.

## Results

There were significantly more females (77%) in the MO group than in the HC group (56%), *p* = 0.0002). The differences in age (*p* = 0.171) and education (*p* = 0.055) were small and not statistically significant. No significant differences were found on test performance scores between MOs with versus MOs without depressive symptoms. The MO patients were therefore handled as one group in the further analysis.

As illustrated in Table 4, there was noticeable variability on the standard RCFT Copy and the Q-score for both MOs and HCs. Upon further inspection, however, the scores were deemed to have acceptable normal distributions, following the recommendations of Montgomery and Runger (2014). On the standard RCFT Copy, 31.3% of the MO and 26% of the HC group performed above the 16th percentile. Although no significant differences in performance between the MOs and HCs emerged on the Copy trial (mean −0.302, CI −1.374 to 0.769, *p* = 0.579), the groups differed on the Q-score (mean −1.784, CI −3.237 to −0.331, *p* = 0.016), though the effect size was small (Cohen's *d* = 0.345). Additionally, the difference between the two groups was statistically significant on the Unit points (mean −1.409, CI −2.291 to −0.528, *p* = 0.002), with a relatively high effect size (Cohen's *d* = 0.451) but not on the Order points (mean −0.351, CI −0.994 to 0.293, *p* = 0.284). The level of differences for the Unit points and the Q-score were slightly reduced when adjusting for sociodemographic factors (see Table 5).

**Table 4:** Performance raw scores on the RCFT for the MO and HC groups.

	**MO (*****N*** = **96)**			**HC (*****N*** = **100)**			
	**Min**	**Max**	**Mean (SD)**	**Min**	**Max**	**Mean (SD)**	**Cohen's *d***
Rey Copy	17.5	36.0	30.19 (3.9)	17.5	36	30.5 (3.69)	−0.08
Unit points	5	17	10.02 (3.26)	7	17	11.43 (2.99)	−0.45
Order points	0	7	1.39 (2.28)	0	7	1.74 (2.26)	−0.15
Q-score	5	24	11.4 (5.3)	7	24	13.18 (5.01)	−0.34
Q-score ^a^	31.49	71.34	48.85 (10.19)				−0.11

a *Q-score standardized as T-score based on the HC*.

**Table 5:** Linear regression analysis, coefficient B for MO (vs HC).

**Dependent variable**	**B**	**CI**	***p*-value**
Rey Copy	−0.302	−1.374 to 0.769	0.579
Adjusted for sex	0.094	−0.961 to 1.149	0.861
Q-score	−1.784	−3.237 to −0.331	0.016
Adjusted for sex	−1.643	−3.102 to −0.184	0.027
Order score	−0.351	−0.994 to 0.293	0.284
Adjusted for sex	−0.307	−0.951 to 0.336	0.348
Unit points	−1.409	−2.291 to −0.528	0.002
Adjusted for sex	−1.325	−2.210 to −0.440	0.004
Q-score^a^	−0.450	−0.842 to −0.058	0.025
Adjusted for sex	−0.457	−0.853 to −0.060	0.024

*N = 96*.

a *Q-score standardized as T-score*.

A closer look at the performance quality exhibited by the MO group revealed that only 28 of the 96 patients (29.1%) started the copying process by completing the base rectangle using four consecutive lines. Completing either the diagonals or bisectors with just two consecutive lines per unit, immediately following the completion of the base rectangle, was carried out by 19 (19.8%) of the 96 patients. Only 5 patients achieved full points on the Order score, and 68 (70.8%) of patients achieved no Order points whatsoever. For the Unit score, only 4 patients achieved maximum points, with 17 successfully completed units. The modal number of successfully completed units was 8 (18.8% of the patients), followed by 9 units (14.6%), and 7 units (11.5%). Overall, the drawing process, as viewed on the RCFT videos of the MO patients, was notably chaotic, sometimes impulsive, and with little attention to neatness, even though they were urged during the instructions to copy the figure as carefully as possible. Unfortunately, this kind of data was not available for our HC because of the RCFT being administered by others than the first author of this study.

When exploring the association between the standard RCFT copy score and the Q-score, a statistically significant difference emerged when analyzing the two groups separately. For the HCs, the Q-score correlated significantly and positively with the standard RCFT Copy score (*r* = 0.228, *p* = 0.023), but that was not the case for the MO group (*r* = 0.165, *p* = 0.109). For the MOs, the performance accuracy score was significantly correlated with the Unit score (*r* = 0.229, *p* = 0.025) which is a sub-score of the total Q-score.

There was a significant negative correlational association between BDI-II and the RCFT Copy (*r* = −0.214, *p* = 0.037), but not between BDI-II and the Q-score (*r* = −0.06, *p* = 0.569). SBP was positively associated with the Q-score (*r* = 0.252, *p* = 0.014), but not the RCFT copy score (*r* = −0.199, *p* = 0.054). Waist circumference did not correlate significantly with any of the RCFT measures for the MO, however, BMI correlated significantly with the standardized Q-score (*r* = −0.239, *p* = 0.025). See Table 6 for a complete correlation matrix.

**Table 6:** Correlation analysis between clinical and neuropsychological variables.

	**Weight**	**BMI**	**Waist circumference**	**WHR**	**Systolic BP**	**Diastolic BP**	**BDI-II**	**RCFT-Copy**	**Q-Score**	**Unit score**	**Order Score**
Weight	1	0.785^**^	0.757^**^	0.309^**^	−0.047	0.107	−0.110	0.047	−0.141	−0.134	−0.136
		0.000	0.000	0.006	0.671	0.327	0.310	0.665	0.190	0.213	0.206
BMI		1	0.615^**^	−0.007	−0.188	−0.060	0.020	0.080	−0.155	−0.146	−0.153
			0.000	0.953	0.083	0.580	0.852	0.458	0.149	0.176	0.155
Waist circumference			1	0.487^**^	0.033	0.096	−0.179	0.069	−0.058	−0.056	−0.055
				0.000	0.762	0.371	0.094	0.519	0.585	0.598	0.606
WHR				1	0.218^*^	0.166	−0.186	0.020	−0.038	−0.019	−0.062
					0.045	0.128	0.087	0.857	0.724	0.862	0.567
Systolic BP					1	0.619^**^	−0.108	−0.221^*^	0.242^*^	0.211^*^	0.261^*^
						0.000	0.306	0.034	0.019	0.043	0.011
Diastolic BP						1	−0.193	−0.119	0.167	0.134	0.198
							0.065	0.254	0.109	0.200	0.058
BDI-II							1	−0.214^*^	−0.060	−0.028	−0.100
								0.037	0.561	0.789	0.333
RCFT Copy								1	0.165	0.229^*^	0.055
									0.109	0.025	0.592
Q-Score									1	0.970^**^	0.937^**^
										0.000	0.000
Unit score										1	0.824^**^
											0.000
Order score											1

*Values are expressed as correlation coefficients (p)*.

* *Sign. at the 0.05 level two-tailed*.

** *Sign. at the 0.01 level two-tailed*.

*BMI, Body Mass Index*.

*WHR, Waist-to-hip ratio: Critical men 0.9 women 0.85*.

*BP, Blood pressure*.

*BDI-II, Beck Depression Inventory II*.

## Discussion

This study presents evidence supporting the presence of inefficiency in visuospatial constructional ability among MO patients. At first glance, the MO patients completed the figure in a satisfactory manner using the standard quantitative scoring for RCFT. The efficient quality of their performance, as reflected by the Q-score process, was significantly below that of those of HCs, however. For the HC group, Q-score performance scores were approximately normally distributed. In contrast, the majority of MO group members scored at the lower end of the HC Q-score performance distribution—even though their Copy trial was complete, accurate, and scored in the normal range based upon the standard accuracy scoring criteria.

**Figure 2:**
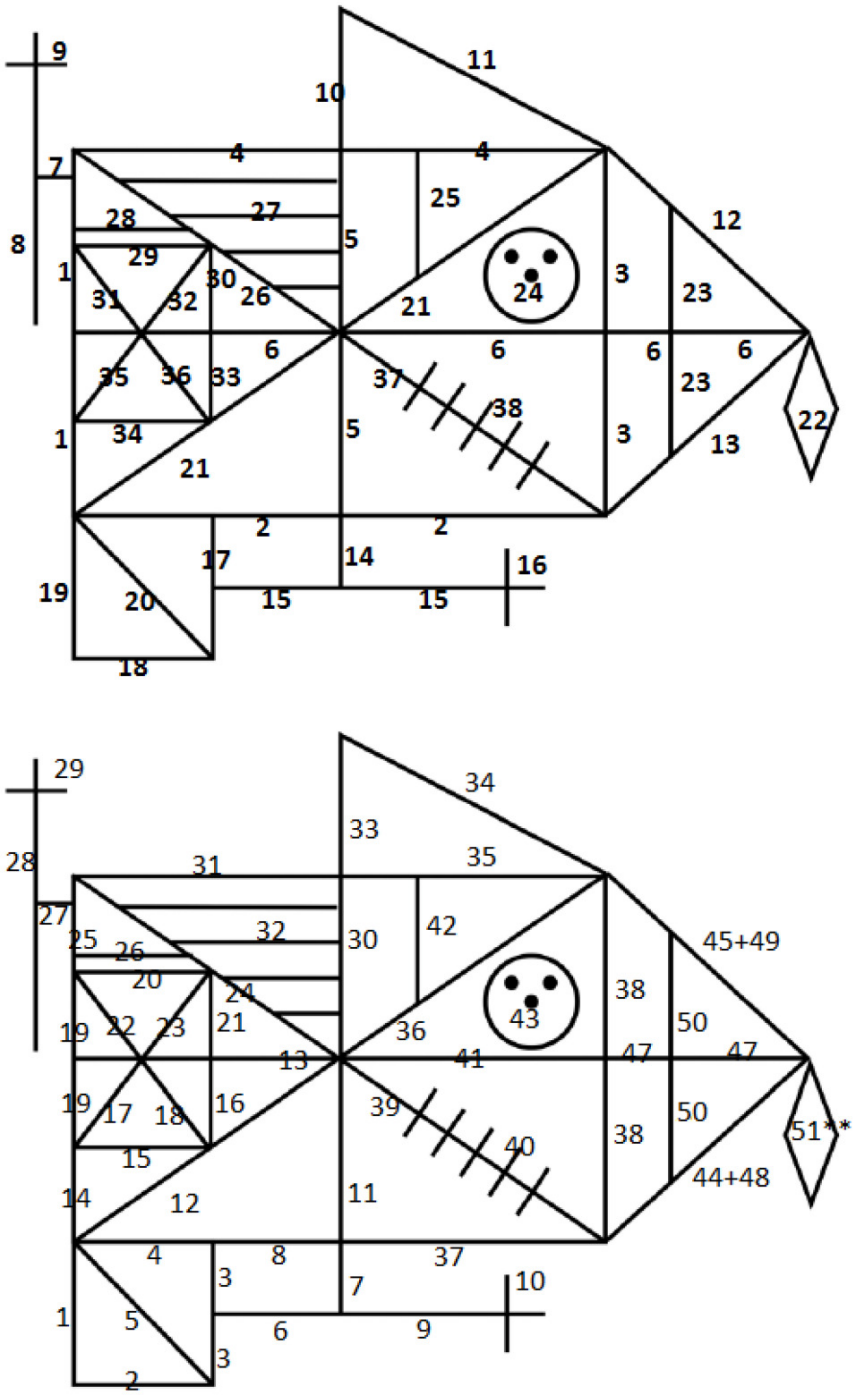
An illustration of the discrepancy between traditional scoring criteria and the Q-Rey scoring system. For the copy trial, the maximum performance accuracy raw score = 36, and for the Q-score = 24. **(Top)** Patient A has achieved an accuracy raw score of 36, and a Q-score of 8. **(Bottom)** Patient B has achieved an accuracy raw score of 23.5, and a Q-score of 19. Patient B has achieved a high Q-score because of an ability to perceive the figure being divided into larger units and has started the copy process with the preferred units first. Patient A has not managed to see the figure as consisting of different units, and has not managed to start with the most important units first when beginning the task.

The discrepancy between the standard RCFT accuracy scoring system and the Q-score became even more evident when we examined differences within the MO group. Those MO patients who earned a maximum score on standard RCFT Copy did not necessarily achieve a good Q-score, and vice versa. Patient A might produce a generally accurate copy with all lines, angles, and proportions correctly drawn, and thereby achieve a top score (See Figures 1, 2), yet when the Q-score scoring criteria were applied, the organizational strategy and efficiency of the drawing process could appear erratic. In contrast, Patient B might achieve a lower accuracy score on the RCFT copy, but achieve a respectably high Q-score, even though the final copy was completed with lines and angles somewhat askew, and the different units were imperfectly proportional to each other. The Q-score would reveal that that patient had completed certain features as specific units, and had therefore approached the task more efficiently, though not accurately.

Typically, individuals should process the RCFT figure at a gestalt level, which would enable them to see the overall picture—perhaps a visual analog of the gist of a story. Instead, the MO patients seem to focus on inspecting the smaller details in isolation rather than those details in the context of the overall figure. Clearly, a few individuals in the MO group struggled with both Unit and Order aspects of the Q-score. It became evident, however, that although the Order points would vary, the deciding factor in overall outcome was the Unit points, suggesting the MO patients struggle to process the smaller units as “building blocks” and perceive their importance in the overall picture in the same way the bigger units of the stimulus figure are perceived. The long-standing view that healthy adults tends to first automatically process the overall features of a visual scene and then fill in the smaller details subsequently, has been challenged though. In fact, recent work has found healthy adults to utilize more piecemeal organizational strategies with considerable fragmentation of the main structural elements than originally thought when completing the RCFT (Wilson and Batchelor, 2015). This undermines the assumption that the stimulus figure should be perceived as meaningful perceptual units with the copying reproduction organized around the base rectangle. Nonetheless, for healthy adults, the best predictor for RCFT copy organization remains to be whether a person perceives the global features or rather relies on local processing according to Wilson and Batchelor. The authors point out that instead of dismissing previous views, we need to use additional tests of organizational strategy to validate findings derived from the RCFT for clinical use in the future. We propose that the Q-score could be one such aid, although as Wilson and Bachelor point out, norms regarding qualitative aspects of RCFT performance is lacking and more testing across healthy and clinical groups need to be conducted.

Although the Roberts et al. (2007) study did not use the Q-score as their qualitative measure, but instead interpreted performance quality (continuous vs. piecemeal, and global vs. local) based on combined performance on the RCFT and the GEFT, they too found evidence of a more fragmented and detail oriented drawing style in their sample of overweight individuals. The authors commented on that issue because of the detail-focused style demonstrated by the overweight subjects; they had expected to see superiority in a task wherein the participants were asked to locate a hidden shape (detail) within a more complex shape. Slower rather than faster times were observed on this task in the overweight group, and the overweight subjects were less able to identify the target shape than were the HCs. In the eating disorder literature, a bias toward detail rather than the big picture has been related to devotion to calorie counting in anorexic patients, to the detriment of global nutritional health (Roberts et al., 2007). In contrast, obese individuals are found to have visual attention bias toward environmental food cues, even when they had recently eaten to satiation, which may play a role in the development and maintenance of obesity (See Hendrikse et al., 2015, for a systematic review). For severely obese individuals attending weight-loss treatment, it would seem to be crucial for the patient to attend to details of their treatment plan to secure a successful treatment outcome. Nevertheless, the success of the treatment plan requires the patient to keep both an attention to detail and an overview of the bigger picture, never sacrificing one for the other.

It may be that for MO individuals, a distorted visual mental-representation of one's body size, shape, and placement in space results from an inefficient visual-spatial organizational strategy, and a resulting inability to see oneself accurately in the complete environmental gestalt. That, coupled with the attentional bias for food cues, may result in a greater emphasis on food consumption, and a disregard of consequent weight gains. In underweight patients, research indicates the lack of improvement in visual-constructive functioning after nutritional rehabilitation, suggesting that the visual-construction difficulties may be antecedent the development of the eating disorder—and failure to improve visual-spatial could signal poor treatment prognosis and contribute to the relapse/exacerbation of the disorder, if not addressed early in the disease process (Alvarado-Sánchez et al., 2009; Rose et al., 2014). In relation to obesity, in contrast, “size blindness”—a distorted visual mental-representation of one's body size, shape, and placement in space—could lead to an accumulation of unwanted excess weight, thereby contributing to worse long-term outcomes. Premorbid functioning level is considered a possible explanatory factor for obesity, with anxiety and depression being considered important antecedents to obesity, and a comorbidity following the onset of obesity.

Habits are closely linked to eating and memory, and much of the treatment effect will be lost if clinicians are not sufficiently attentive to a patient's detrimental habits and misapprehensions of facts when the treatment begins. Most people often engage in unconscious eating (eating without thinking). All surrounding stimuli or environmental factors—which may appear unrelated to the behavior itself—are influencing consumption volume by inhibiting self-monitoring (Wansink, 2004). As time passes, these environmental factors have alternated consumption norms, and the associations between the stimuli/cues and the response have become automatized. For instance, bariatric surgery patients have been documented as primarily complying with their eating plan the first 6 months post-surgery. After that, however, many patents start experimenting with food volume and forbidden high-caloric food, and the frequency of snacking increases (Bochieri et al., 2002). Some patients even return to their old eating patterns (Glinski et al., 2001). Inaccurate perception may be a barrier to motivational and behavior change necessary for weight reduction (Adams et al., 2006). Patients who believe that they are not as big as the other patients in the clinic, who do not perceive the extent of their problem, are unlikely to be motivated to undertake the behavior change that would allow them to lose weight.

Our study does not determine exactly how impaired visual-constructional ability dominated by local processing affects treatment outcome. Poor organization of local processing is well known to have detrimental effects on memory, however. Excess body weight is found to impact learning, and in turn, certain memory processes. It is possible that obese individuals have a reduced ability to connect spatial location and object identity into coherent and vivid memories when presented with a visual task (Cheke et al., 2016).

We propose that the Q-score, through its unique approach of assessing for both order of production and the identification of composite unit components, is better suited for capturing the hidden nuances of the way subjects encode information. The combined use of Unit and Order points in the calculation of the Q-score makes this qualitative scoring system more favorable compared to other scoring methods. Additionally, from a clinical standpoint, the Q-score system has clear advantages, in that it is a brief and easy system to use when scoring the RCFT, yet it provides detailed estimation about an individual's degree of efficient planning and strategic approach to a task. Perhaps it would be more beneficial for treatment outcome if this were considered when developing treatment plans, as it could aid clinicians in deciding which “everyday life impairments” should be targeted when facilitating the adoption of healthier habits.

### Strength and limitations

A key strength of our study is the moderate sample size of the MO and HC groups. The sample was adjusted for demographic variables and disease-related factors (depressive symptoms, anthropometrics, and blood pressure), in our analysis of RCFT performance for the MO group. In addition, we used both quantitative and qualitative scoring criteria to assess test performance.

A weakness of our study is the lack of comparable data for clinical factors regarding HC subjects. We did not have any information on how each HC in specific approached the task either. Furthermore, because no data were available on the RCFT recall measures for the HCs, we were unable to use recall data for our MO in our comparative analysis. Therefore, we cannot provide information on the effect of body composition on visual memory. Our study provides only a descriptive for this specific sample, and performance on the Copy trial only. Nonetheless, the purpose of this study was to explore potential associations rather than causality.

### Implications

This study suggests that patients with obesity have difficulties in the way they approach a drawing sequence and how efficiently they encode material that later needs to be retrieved. Additionally, we believe that we have found indication that the Q-score is a good measure for capturing a wider range of cognitive processes, which are not described by traditional scoring methods. This is the first study of its kind involving MO patients.

## Author contributions

HS was responsible for data collection, data analysis, and data interpretation, and wrote the initial draft of the manuscript. FB provided data for the US and Canadian samples. SL was involved in data analysis. KH designed and headed the study. All authors were involved in writing and editing the manuscript and in approving the final manuscript.

### Conflict of interest statement

The authors declare that the research was conducted in the absence of any commercial or financial relationships that could be construed as a potential conflict of interest.
